# The Marine Natural Product Furospinulosin 1 Induces Apoptosis in MDA-MB-231 Triple Negative Breast Cancer Cell Spheroids, But Not in Cells Grown Traditionally with Longer Treatment

**DOI:** 10.3390/md19050249

**Published:** 2021-04-28

**Authors:** Esther A. Guzmán, Tara P. Pitts, Priscilla L. Winder, Amy E. Wright

**Affiliations:** Marine Biomedical and Biotechnology Research, Harbor Branch Oceanographic Institute, Florida Atlantic University, 5600 US 1 North, Ft. Pierce, FL 34946, USA; tpitts3@fau.edu (T.P.P.); pwinder@fau.edu (P.L.W.); awrigh33@fau.edu (A.E.W.)

**Keywords:** marine natural products, triple negative breast cancer, spheroids, high content imaging, apoptosis, furospinulosin 1

## Abstract

Cancer cells grown in spheroid conditions interact with each other and the extracellular matrix, providing a better representation of the in vivo environment than two-dimensional cultures and are a more clinically relevant model. A discrete screening of genetically diverse marine samples in the spheroid assay led to the identification of a novel activity for the known compound furospinulosin 1. This compound shows activity against MDA-MB-231 triple negative breast cancer cells grown as spheroids and treated for 24 or 48 h. No cytotoxicity was seen in traditional two-dimensional adherent cultures treated for a longer time (72 h). A reverse phase protein array (RPPA) confirmed the limited activity of the compound in cells grown traditionally and revealed changes in protein expression when cells are grown as spheroids that are associated with better clinical prognosis. Analysis of the RPPA data through the Broad institute’s connectivity map suggested the hypothesis that furospinulosin 1 functions as an MEK inhibitor. Analysis of the RPPA data through STRING supports the apoptosis observed. The selectivity exhibited by furospinulosin 1 for triple negative breast cancer cells only when grown as spheroids makes it an interesting compound with strong therapeutic potential that merits further study.

## 1. Introduction

The American Cancer Society estimates that 284,200 people (~1% patients are males) will be diagnosed with breast cancer in 2021, with an estimated 44,130 deaths expected from this disease [1]. Improvements in early detection as well as the advent of targeted therapies have considerably reduced the mortality of breast cancer. However, triple negative breast cancers—the collective term for breast cancers that do not express the estrogen receptor (ER) and the progesterone receptor (PR), and do not overexpress the human epidermal growth factor receptor type 2 (HER 2)—are resistant to most types of targeted therapies. About 10–20% of breast cancer diagnoses in the US are of triple negative breast cancer [1]. These cancers are more prevalent in black women than white women, tend to affect pre-menopausal women and are associated with a BRCA1 gene mutation [1]. A high incidence of triple negative breast cancer has also been found in Mexican women; in this population, about 25% of those diagnosed with TNBC carry a BRCA1 mutation [2]. Triple negative breast cancers can aggressively spread to other organs, particularly the brain and the lungs [3], and are more likely to recur than other breast cancers [4]. They are labeled as high-grade tumors because the cells bear very little resemblance to normal cells [3,4]. Triple negative breast cancer is sometimes described as being basal type, based on the resemblance of these cancer cells to the basal cells that line the breast ducts, although the cancer does not necessarily arise from these cells [2,3]. Triple negative breast cancer also encompasses the cloudin-low tumors, which have properties similar to those of stem cells [3]. The MDA-MB-231 cell line used in this study has been classified as cloudin-low, while the MDA-MB-468 cell line also used in this study has been classified as basal [5]. MDA-MB-231 has mutations in BRAF, CDKN2A, KRAS, NF2 and TP53, while MDA-MB-468 has mutations in PTEN, RB1, SMAD4 and TP53 [6]. There is no preferred standard form of chemotherapy for TNBC, although paclitaxel has shown benefits [3]. The drug ixabepilone, which is a synthetic analogue of the natural product epothilone B, shows promise in the treatment of TNBC [4]. TNBC is a collection of different kinds of cancers rather than a single form of the disease, which may be the reason why finding targeted therapies against TNBC remains a challenge for researchers [2].

The current paradigm of drug discovery encompasses the screening of compound libraries in 2D cell cultures, with those compounds that induce cytotoxicity in 2D models being studied in animal models, and then if successful, taken to clinical trials. However, only about 10% of compounds identified through a 2D screen are effective in clinical trials [7]. This lack of a response is in part attributed to the limitations of screening cells grown in 2D, as studying cells in isolation does not adequately reflect the environment that most tumors find themselves in, where they are surrounded by the extracellular matrix and other cells that activate signaling pathways and provide soluble factors that help create the microenvironment [7]. Three-dimensional cultures are an established model that is finding resurgence as studies using 3D cultures appear to be more clinically predictive [7,8,9]. Three-dimensional cell cultures develop chemical gradients where oxygen, nutrients and catabolites (to name a few) are present at different levels in different parts of the spheroid [8]; these gradients are more apparent in larger spheroids. Cells in spheroids also contain different phenotypes with quiescent, necrotic, proliferating and hypoxic cells present within a spheroid [7]. Cells in a 3D culture environment show differences in gene, protein and cell receptor expressionwhen compared to their 2D counterparts [7]. Not surprisingly, very different responses are seen when evaluating potential chemotherapeutics using a 2D vs. a 3D cell culture [7,8,9]. While in an ideal world drug discovery would be performed by testing on animals first, this is neither cost effective nor ethical. Three-dimensional cultures provide a compromise between animal testing and 2D cell cultures, with a higher cost but enhanced predictability. The use of 3D cultures in drug discovery is becoming a new paradigm [10,11].

The utility of natural products to medicine is undeniable [12]. Forty-nine percent of current cancer treatments originate from natural products [13]. Oceans cover over 70% of the Earth’s surface and contain a wealth of biological diversity with more than 200,000 described species of marine invertebrates and algae and millions of microbial species. It is likely that there is an equal number yet to be discovered. In the past seven years, more than 7000 new compounds have been reported from marine organisms. These compounds encompass a wide variety of chemical structures, including acetogenins, polyketides, terpenes, alkaloids, peptides and many compounds of mixed biosynthesis. A number of excellent reviews document the diversity of both structures and bioactivities which have been observed for marine-derived compounds [14].

The uniqueness, chemical diversity and structural complexity of marine natural products represent an unexploited supply of potential new drugs, lead compounds for medicinal chemistry or biological probes to allow for a better understanding of diseases. The Marine Biomedical and Biotechnology Research Program at HBOI has developed a unique library of pure compounds and highly enriched fractions derived from marine organisms that were evaluated in this study. Over the past year, high content imaging (HCI) of spheroids has been implemented into our screening program. The triple negative breast cancer cell lines used, MDA-MB-468 and MDA-MB-231, show an invasive phenotype in vitro and are tumorigenic in vivo [5] and grow in tumor spheroids using low adherence plates in the presence of 2.5% matrigel [15,16]. Cancer cells grown in spheroid conditions (3D cultures) allow the cells to interact with each other and the extracellular matrix, providing a better representation of the in vivo environment than two dimensional adherent cultures. Therefore, results obtained using this model are more clinically relevant [7,8,9,10,11].

A discrete screening of genetically diverse marine samples in the spheroid assay led to the identification of a novel activity for the previously reported compound furospinulosin 1. We report herein the activity of furospinulosin 1 against the MDA-MB-231 triple negative breast cancer cells when grown as spheroids but not when grown in traditional two-dimensional adherent cultures.

## 2. Results 

### 2.1. Screening Assay Results

Cells were plated on a 384-well low adherence plate and incubated for 24 h to allow for the formation of a spheroid. Spheroids were then treated for 48 h, at which point they were assessed using the multiparametric assay described above. Compounds that decrease total cell counts by 50% or more, compounds that increase caspase cleavage by 30% or more, or compounds that show an increase in cell permeability of 30% or more were considered hits. All actives were repeated to confirm hits. Fractions from the enriched fraction library were tested at 5 μg/mL; this concentration was selected as it often leads to compounds with activity in the low to mid nM range after final purification of the active compounds.

**Figure 1:**
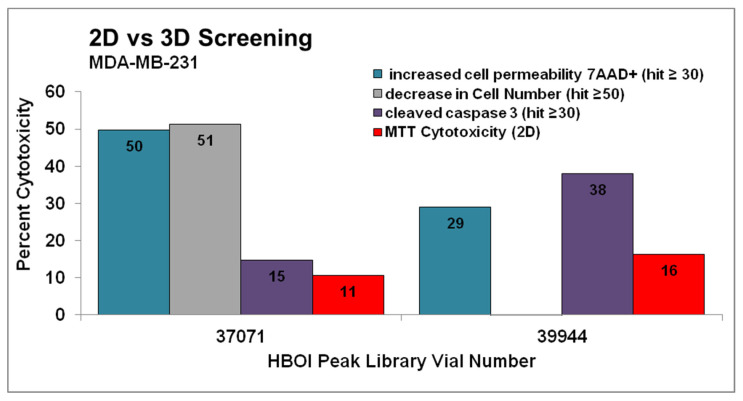
Fractions 37071 and 39944 were chosen as hits in a multiparametric assay to determine cytotoxicity in MDA-MB-231 cells grown as spheroids. Compounds that decreased total cell levels by 50% or more (grey columns), compounds that increase caspase cleavage by 30% or more (purple columns), or compounds that show an increase in cell permeability of 30% or more (teal columns) after 48 h treatment were considered hits, and were confirmed by repeating the testing. These results are shown next to cytotoxicity in the same cell line tested at 72 h in cells grown as adherent monolayers (red). Both of these fractions were active in the spheroid assay but would not have been hits in the MTT assay. Fractions were tested twice in the spheroid assay and one time in the MTT assay.

Fractions 37071 and 39944 from marine sponges of the order Dictyoceratida were selected as hits in the spheroid assay in the MDA-MB-231 cell line. They were not deemed hits in the MDA-MB-468 cell line. These samples were also tested in a standard cytotoxicity assay (MTT). For the latter, cells were plated, allowed to adhere overnight, and then treated for 72 h with the same samples at the same concentrations used in the spheroid assay. The results for these samples in the MDA-MB-231 spheroid assay were graphed along with their 2D cytotoxicity (Figure 1). Both fractions 37071 and 39944 exhibited very modest cytotoxicity under 2D culture (red in Figure 1) while meeting at least one of the criteria for a hit in the spheroid assay. These fractions were also tested in MDA-MB-468 cells in this assay, but did not exhibit similar activity.

### 2.2. Identification of the Compound Responsible for the Activity

**Figure 2:**
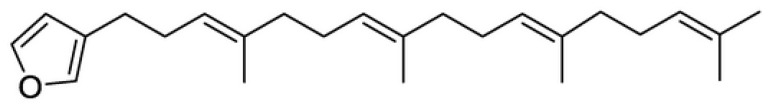
Structure of furospinulosin 1.

After chemical evaluation, these fractions were shown to contain different concentrations of the known compound furospinulosin 1, whose structure is shown in Figure 2. Fraction 39944 was further fractionated to provide pure furospinulosin 1 used in the remainder of the studies.

### 2.3. Determination of Concentration Required to Obtain 50% Activity

**Figure 3:**
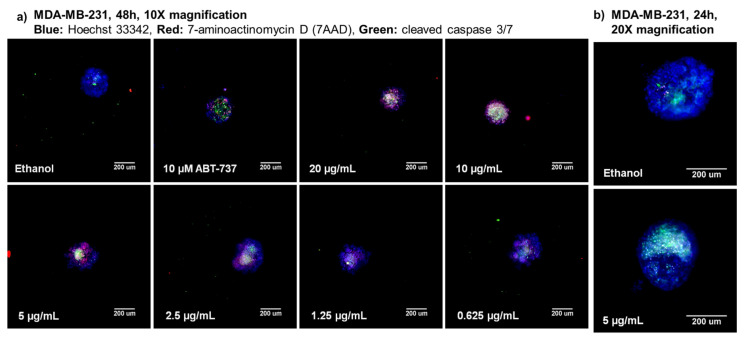
3D spheroid high-content images for furospinulosin 1. (**a**) TNBC MDA-MB-231 cells were plated at a density of 1500 cells per well, and a spheroid was allowed to form overnight. The next day, cells were treated for 48 h with serial dilutions of furospinulosin 1 ranging from 20 to 0.156 μg/mL; ethanol (solvent control); or ABT-737, a known drug control. After treatments, cells were labeled with the cell permeable nuclear stain Hoechst 33342 (blue), a marker for cleaved caspase 3/7 (green) and the cell impermeable nucleic acid stain 7AAD (red) for three hours. Cells were fixed, and images were acquired at 10× magnification and analyzed using HCI. Experiment was repeated 3 times, with one representative set of images shown. (**b**) To show the spheroids in more detail, MDA-MB-231 cells were grown as spheroids overnight then treated with 5 μg/mL furospinulosin 1 or vehicle control for 24 h and stained as described above. Spheroids were imaged at 20× magnification using HCI. Experiment was performed once.

MDA-MB-231 cells were treated with serial dilutions from 20 to 0.156 µg/mL of furospinulosin 1 for 48 h in the spheroid screening assay to confirm it was responsible for the activity seen in the fractions. Cytotoxic activity was seen with this compound starting at a concentration of 0.625 µg/mL but not achieving our criteria for a hit until the 5 µg/mL concentration (Figure 3a). Images were acquired using a 20× objective to show the spheroids in more detail from a single experiment when MDA-MB-231 cells were treated with 5 µg/mL of furospinulosin 1 or vehicle control for 24 h (shown in Figure 3b).

**Figure 4:**
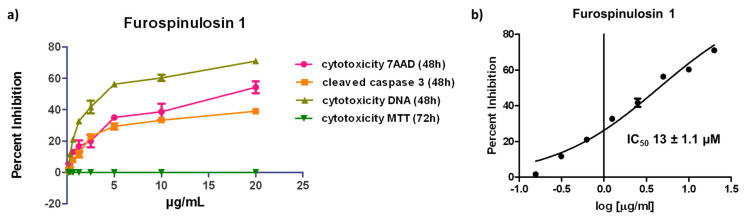
(**a**) 3D Spheroid and 2D cytotoxicity data for furospinulosin 1. Cells were grown overnight then treated for 48 (spheroid assay) or 72 h (MTT assay) in duplicate wells with serial dilutions of furospinulosin 1 ranging from 20 to 0.156 μg/mL or ethanol (solvent control). The data were normalized to vehicle control, and the average of 3 experiments shown as cytotoxicity was graphed using Graph Pad Prism. (**b**) IC_50_ for furospinulosin 1 in the spheroid assay is 13 ± 1.1 µM. The data for cell decrease based on Hoechst 33342 staining (cell decrease) in the spheroid assay were normalized to vehicle control and subjected to a non-linear regression curve fit analysis using Graph Pad Prism. The values shown are the average of 3 experiments ± standard deviation.

MDA-MB-231 cells were also treated using serial dilutions from 20 to 0.156 µg/mL of furospinulosin 1 in the traditional MTT assay. Furospinulosin 1 failed to induce any cytotoxicity at any of the concentrations tested, meaning that the IC_50_ for cytotoxicity at 2D is >20 µg/mL (≈56 µM). The average results from the three experiments in the MTT assay were graphed (dark green; on top of x-axis) next to the average results of the three experiments in the spheroid assay in Figure 4a.

The data for overall cell decrease based on the cell-permeable nuclear stain Hoechst 33342 were subjected to a non-linear regression curve fit analysis to obtain an IC_50_ for cytotoxicity in the spheroid assay for furospinulosin 1 of 13 ± 1.1 µM (Figure 4b). The values shown represent the average ± standard deviation from three independent experiments.

### 2.4. Differential Protein Expression Analysis

MDA-MB-231 cells were grown in 2D monolayers or as spheroids (3D) overnight and treated with ethanol (vehicle control) or 5 μg/mL furospinulosin 1 for 24 h. Protein was isolated from these samples and subjected to a reverse phase protein array containing 450 antibodies at MD Anderson Cancer Center RPPA core. These antibodies were selected to represent key signaling pathways including regulators of immune responses, apoptosis, cell cycle progression, DNA repair and autophagy. The data were analyzed using R statistical software, and both the raw data and a heat map were generated at the core and provided to the user. The normalized linear data received were averaged for the three independent experiments that were run in the same array, and the standard error of the mean was calculated. For every comparison made, the function of the five proteins most upregulated and the five most downregulated is summarized in a table provided in the supplementary information. The information in the table was obtained from the Human Protein Atlas [17], Online Mendelian Inheritance in Man^®^ (OMIM) [18] or the antibody provider Cell Signaling Technologies (Danvers, MA, USA).

**Figure 5:**
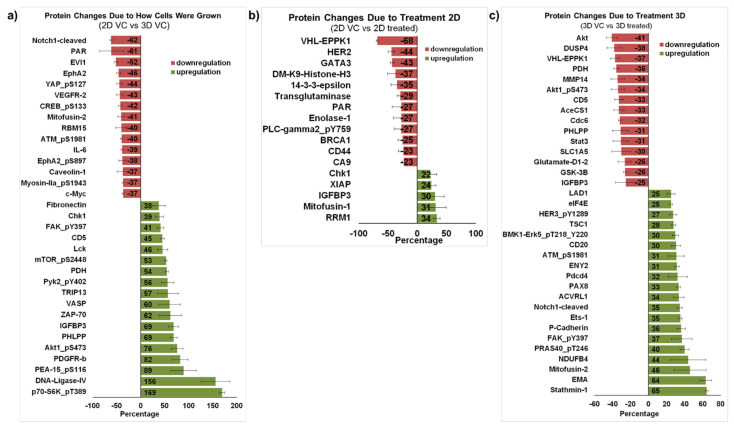
Proteomic profiling of MDA-MB-231 cells treated with furospinulosin 1. (**a**) Protein changes due to how cells were grown. The average linear normalized data for cells grown as spheroids (3D) and treated with ethanol (vehicle control) for 24 h was further normalized to results from cells grown traditionally (2D) treated with vehicle control for 24 h. The average of 3 independent experiments ± standard error of the mean is expressed as percentage decrease/increase. Proteins with greater than 35% change and whose error bar was ≤half the value are shown. (**b**) Protein changes due to 2D treatment. The average linear normalized data for cells treated with furospinulosin 1 were compared to those of cells treated with ethanol (vehicle control) for cells grown traditionally (2D) and treated for 24 h. The average of 3 independent experiments ± standard error of the mean is expressed as percentage decrease/increase. Proteins with greater than 20% change and whose error bar was ≤half the value are shown. (**c**) Protein changes due to 3D treatment. The average linear normalized data for cells treated with furospinulosin 1 were compared to those of cells treated with ethanol (vehicle control) for cells grown as spheroids (3D) and treated for 24 h. The average of 3 independent experiments ± standard error of the mean is expressed as percentage decrease/increase. Proteins with greater than 25% change and whose error bar was ≤half the value are shown.

The differential protein expression of solvent control-treated cells grown in 2D monolayers compared to those grown as spheroids is shown in Figure 5a. Only proteins with a change greater than 35% with error bars that were less than or equal to half the change are shown. This figure highlights expected changes in protein expression due to the way the cells were grown. As many groups have previously reported, it is obvious that changes in signal transduction are caused by growing the cells as spheroids rather than in the more traditional 2D-monolayer method.

Figure 5b compares MDA-MB-231 cells grown traditionally treated with solvent control against those treated with 5 μg/mL furospinulosin 1. Only proteins that changed more than 20% with error bars less than or equal to half the change are shown. While furospinulosin 1 does not induce cytotoxicity in the cells grown in 2D at the time and concentrations tested, it downregulated both HER2 (44%) and BRCA1 (25%) proteins in these cells. The downregulation of BRCA1 was also seen in 3D-treated cells, but it was only 6% (data not shown). Few changes were observed in this comparison, which is in agreement with the lack of cytotoxicity seen in 2D grown cells.

Protein from MDA-MB-231 cells grown as spheroids and treated with vehicle control was compared to protein from spheroids treated with 5 μg/mL furospinulosin 1. Proteins that changed more than 25% with error bars less than or equal to half the change are shown in Figure 5C, which highlights the different proteins affected by furospinulosin 1 treatment in the 3D spheroids. The protein with the greatest decrease was Akt (41%), a well-known inhibitor of apoptosis [19]; its phosphorylated form at serine 473 (Akt1 pS473), which is necessary for AKT1 activity, also exhibited a 34% decrease. The PI3K/mTOR/AKT pathway is considered a therapeutic target for triple negative breast cancers [20,21,22]. In a small study that compared primary and metastatic breast cancer tumors, GSK3β emerged as one of the genes differentially expressed between the two types of tumors, and its expression was associated with shorter overall survival [22]. The treatment of spheroids with furospinulosin 1 reduced its expression by 26%. Pyruvate dehydrogenase (PDH), which was decreased about 36% by furospinulosin-1 in spheroids, is known to facilitate tumor progression by affecting the metabolic state of cancer cells activating glycolysis; silencing its expression inhibits tumor growth [23]. In addition, the treatment of spheroids with furospinulosin 1 decreased the signal transducer and activator of transcription 3 (STAT3) by about 33%. STAT3 downregulation is a desirable outcome in cancer cells, as this transcription factor regulates inflammatory and metastatic pathways, has been associated with immune evasion and is a therapeutic target for breast cancer [24]. Treatment with furospinulosin 1 increased the expression of Stathmin 1 by about 65% percent. Stathmin 1 is an oncoprotein that is associated with negative prognosis in patients with breast cancer [25]. It regulates microtubule dynamics based upon its phosphorylation state [26]. In the non-phosphorylated state, it binds the tubulin dimer to form the T2S complex, thereby sequestering tubulin and making it unavailable for cell division. It also binds the growing tips of microtubules acting to de-stabilize microtubules and induce catastrophe. When phosphorylated, it loses tubulin binding efficiency, freeing tubulin for cell division [26] and, in cancer cells, its phosphorylation and dysregulation have been shown to occur through the activation of Raf kinase [27]. Furospinulosin 1 treatment increased Mitofusin-2 expression by 46%. In triple negative breast cancers, mitofusin-2 is a catalyst of mitochondrial fusion, which results in cell cycle arrest and the inhibition of cell proliferation [28]. Similarly, furospinulosin 1 treatment increased the epithelial membrane antigen (EMA, MUC-1 and episialin) by 64%. The expression of this mucin-like transmembrane glycoprotein has been associated with increased overall survival and increased relapse-free survival in patients with ductal breast cancers [29,30].

### 2.5. Bioinformatic Analysis of Proteomic Results

**Figure 6:**
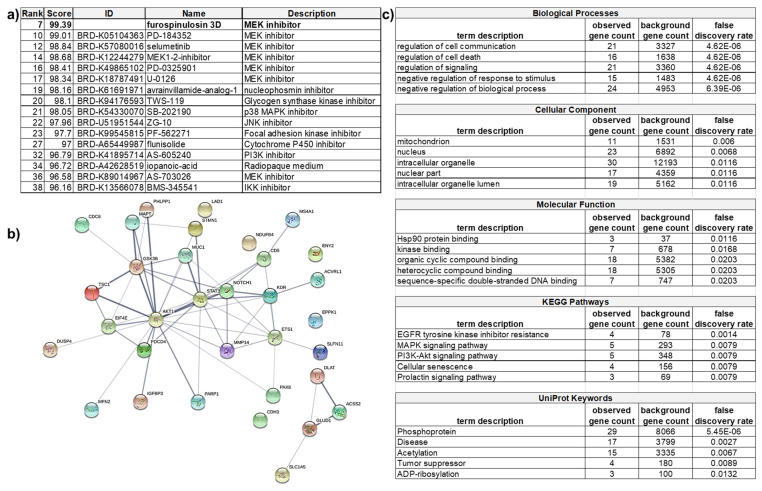
Analysis of RPPA data in the Connectivity Map and STRING databases. Proteins that were changed at least 25% from cells treated with furospinulosin 1 compared to vehicle control treated cells that were grown as spheroids were analyzed in these databases. (**a**) Connectivity Map compares the profile to known compounds and suggests that the mechanism of action (MOA) of furospinulosin 1 may be MEK inhibition. (**b**) STRING looks at protein interactions and (**c**) shows that furospinulosin 1 has effects on apoptotic signaling and signal transduction. This supports the effects seen and neither proves nor disproves the hypothesized MOA obtained from the connectivity map.

The averaged results from the protein array for the 3D spheroid-treated cells were entered into the Broad Institute’s Connectivity Map. The connectivity map contains over a million genetic profiles generated using small molecules, shRNA, cDNA and biologics [31]. This Next Generation Connectivity Map is about a 1000-fold scale up of the connectivity map created as part of the NIH Library of Integrated Network-Based Cellular Signatures (LINCS) consortium [31]. This new connectivity map has shown utility in discovering the mode of action of small molecules, although they estimate that only 63% of small molecules show a correlation to their cellular targets [31]. However, it is a useful tool to formulate hypotheses as to what the potential mode of action of a compound could be. As the connectivity map uses genomic profiles, all data related to protein modifications, such as phosphorylation, were eliminated. Similarly, since only a list of the most upregulated and downregulated proteins without using a value was entered, only those proteins with a difference greater than 25% compared to vehicle control were used. Based on this, a list of compounds with similar patterns was obtained, resulting in the hypothesis that furospinulosin 1 could be acting as an MEK inhibitor (Figure 6a). MEK inhibitors have shown promise in the treatment of triple negative breast cancer, although resistance appears to be an issue; this, however, can be potentially overcome by the use of the right drug combination [32]. This hypothesis prompted a closer look at the proteins included in the array that correspond to the MEK pathway. While there are proteins involved in transcriptional regulation downstream of phosphorylated Erk 1/2 that are downregulated, not all of the proteins downstream are downregulated (Supplemental Figure S6). Furthermore, among the most downregulated proteins is DUSP4, which is part of the negative feedback of Erk 1/2 [33], and downregulation should lead to Erk 1/2 being more active. On the other hand, MEK inhibitors are known to inhibit the phosphorylation of Stathmin1, leading to the block of cell proliferation and the induction of apoptosis [27]. Further investigation is needed to confirm that the inhibition of MEK is the mode of action of furospinulosin 1. Due to the strong changes in Akt caused by furospinulosin 1, it is possible that the regulation of the Erk pathway is mediated through its crosstalk with the Akt pathway [34].

The subset of proteins that changed more than 25% in 3D-treated cells was also analyzed through the Search Tool for the Retrieval of Interacting Genes/Proteins (STRING) database to look at the functional interactions of proteins [35]. As the subset was rather small, only a normal gene set analysis was performed rather than a ranked-based testing. The ranked-based testing would use the value of how much a protein was overexpressed or downregulated in its search for functional enrichment. However, this type of analysis requires large gene lists, such as whole genomes. A gene set enrichment analysis seeks to identify a functional profile of the protein set entered by using statistical methods to identify significantly enriched or depleted groups of proteins. It does not take into account the percentage that the protein was upregulated or downregulated. STRING created a network from the proteins in the subset. This network included 33 nodes (proteins) and 57 edges (lines denoting associations). From these many nodes, the expected number of edges was 22, suggesting that the proteins had more interactions than expected. The p value for the enrichment was 3.21 × 10^−10^, which suggests that the proteins in the network are biologically connected as a group (Figure 6b; an enlarged image is also included as Supplementary Figure S7). In this network, the lines denote an association: the darker the line the higher the confidence. Of interest, AKT1 seemed to have many more significant interactions than other proteins in the subset. These associations do not denote direct binding, but rather, they may contribute to a shared function. Other proteins, such as epiplakin (EPPK1), showed no associations to any other protein. The network was further analyzed, providing tables that show the functional enrichments for biological processes, molecular function, cellular component, KEGG pathways, UNIprot Keywords, among others. The Kyoto Encyclopedia of Genes and Genomes (KEGG) is a database used to understand the function of a biological system. UNIprot is another database of protein sequences and functional information. The top five functional enrichments for each category listed are shown in Figure 6c. The gene count represents how many proteins in the network are annotated with a particular term. The background gene count represents how many proteins in the whole human genome are annotated with that term. The false discovery rate describes how significant the enrichment is by showing the p values. The signaling cascades affected include apoptosis, which supports the apoptosis seen in the screening assay. Concerning the hypothesized mechanism of action, this analysis neither proves it nor disproves it. Both the MAPK and the PI3K/AKT pathways showed similar enrichment.

## 3. Discussion

This article reports the novel activity of furospinulosin 1 to selectively target MDA-MB-231 TNBC cells only when they are grown as spheroids. While the compound was tested in two TNBC cell lines, it was only selected as a hit against the more stem-cell-like cell line MDA-MB-231 rather than the basal line MDA-MB-468. Given the differences between the cell lines, this result is not entirely surprising. Triple negative breast cancer is often described as a collection of many different cancers with the unifying characteristic of lacking the estrogen and progesterone receptors and not overexpressing Her2. Nevertheless, the activity seen in MDA-MB-231 spheroids suggests that furospinulosin 1 may be able to treat cloudin-low triple negative breast cancers. This finding is also novel in that many anticancer agents have higher IC_50_s against TNBC spheroids than against cells grown in 2D [6]. In a study of the known anticancer drugs epirubicin, cisplatin and docetaxel in thirteen TNBC cell lines grown as spheroids, these drugs consistently had higher IC_50_ in spheroids than 2D cultures [6]. Furospinulosin 1 shows exquisite sensitivity for spheroids, inducing apoptosis and significant protein changes in MDA-MB-231 cells grown as spheroids within 24 h of treatment, while having minimal effects on 2D grown cells with a treatment as long as 72 h.

Furospinulosin 1 is a furanosesterterpene previously isolated from several marine sponges of the order Dictyoceratida, including *Hippospongia* [36], *Ircinia* [37], *Spongia* [38], *Fasciospongia* [39], *Idia* [40] and *Smenospongia* [41] species. The only known biological activities for this compound are cytotoxicity ranging from 105 to 155 μM against colon cancer HCT-116 cells bearing p53 and p21 knockouts as well as the wild-type parent cells [41] and anti-parasitic activity ranging from 32 to 77 μM against protozoans [42]. Of interest, in this latter study, furospinulosin 1 was tested for cytotoxicity against the rat myoblast cell line L6, which is not cancerous and is considered a normal cell line. The cytotoxicity reported was >90 µg/mL (≈254 µM) [42], meaning that no cytotoxicity was seen at that concentration. These data, along with the results reported here where no cytotoxicity was seen in 2D cultures, suggest the selectivity of this compound for triple negative breast cancer spheroids. Greater activity was observed in 3D cultures with a shorter treatment time and no activity in 2D with the longer treatment. In both cases, the results were normalized to their solvent controls, ascribing 100% viability to this control. Thus, any cytotoxicity caused by the compound in either of the assays would have been captured in the overall results. The IC_50_ in the spheroid assay was determined to be 13 µM, highlighting how much more potent the compound is against spheroids than against cells grown in 2D both in the testing reported in this article and in the previously published results described above.

While it is clear that a marked difference in the activity of furospinulosin 1 was seen when the cells are grown in 2D versus 3D, it is not clear why. The marked differences in protein expression and activation caused by how the cells were grown (shown in Figure 5a) could suggest that some of those changes either facilitate or enhance the compound’s activity, perhaps allowing for an interaction between the compound and a signaling pathway that otherwise does not occur in 2D grown cells. Growing the cells as spheroids may also facilitate the entry of the compound into the cells. Finally, the microenvironment created by growing the cells as spheroids where hypoxia and reactive oxygen species are present may cause a change in the compound that enhances its activity. The fact that treatment in 2D led to few changes in protein expression and failed to induce cytotoxicity seems to support this speculation. It is clear that furospinulosin 1 appears to behave in a manner fundamentally different to other cytotoxic compounds that are active in 2D cultures.

Ideally, determining the induction of apoptosis by compounds is performed by using two markers of apoptosis. For this compound, this premise was challenging as all methods would have needed to be adapted to 3D, which is not easy. Since furospinulosin 1 not only caused cleavage of caspase 3 but also affected overall cell number, as measured by DNA content, and caused cells to lose their membrane integrity, as measured by 7AAD staining, there is strong confidence that furospinulosin 1 induced apoptosis in MDA-MB-231 spheroids. This belief is further supported by the STRING analysis, which showed enrichment in the apoptosis pathway in spheroid cells treated with furospinulosin 1.

The proteomic profiling shown is the average of three independent experiments, providing confidence in the protein changes that are shown. The array contains 450 antibodies, and performing this differential proteomic profiling was essential to determine future targets for confirmation. Furthermore, this analysis confirmed how different the cells behave when grown in 2D versus 3D, providing a plausible explanation as to why the compound behaves so differently in 2D versus 3D cultures. The proteomic profiling further confirmed the limited effects seen when treating the cells with furospinulosin 1 in 2D.

The proteomic profiling shown here also allowed the performance of some bioinformatics analysis that led to the hypothesis that MEK inhibition is the mechanism of action of furospinulosin 1 in MDA-MB-231 spheroids. However, the data at hand do not provide strong confidence that this is the case, since not all the proteins downstream of MEK were downregulated in the proteomic profiling, as shown in the Supplemental Figure S6. One of the most downregulated was DUSP4, a negative regulator of Erk, whose downregulation should result in its activation. Moreover, an MEK inhibitor would have also shown activity in 2D, unless the difference in activity relies on some of the changes caused by growing the cells in 3D. These changes could allow for a different interaction of the compound with a protein or signaling pathway that is not possible in 2D, enhance the ability of the compound to enter the cells or even change the conformation of the compound resulting in enhanced activity. The changes caused to Stathmin are of interest given its regulation of microtubules and cell division, and future experiments will be performed to further the understanding of those changes. A current hypothesis based on the proteomic results is that furospinulosin 1 acts on the Akt pathway (suggested by the reduction on expression levels of both the phosphorylated and unphosphorylated protein) and the inhibition of MEK occurs through the crosstalk of the Akt and MEK signaling pathways. Confirmation of the protein targets and the hypothesized mechanism of action that this analysis identified will be the subject of future experiments.

To our knowledge, this compound has not shown any previous activity against breast cancer cells. The induction of apoptosis in MDA-MB-231 triple negative breast cancer cells grown as spheroids is a new activity for this compound. Moreover, this is a compound that would never have been identified if only the traditional 2D cytotoxicity screening was conducted, highlighting that the 3D screening assay performed allows the identification of compounds that would be missed if only screening in 2D cultures was performed. Three-dimensional cultures are an established model that is finding resurgence as studies using 3D cultures appear to be more clinically predictive [6,7,8,9,10,11]. The proteomic analysis performed here appears to support this premise, as most of the proteins that were downregulated or upregulated by furospinulosin 1 in cells grown as spheroids are associated with a better clinical prognosis. There were, however, changes that were unexpected, which supports performing further studies with this molecule. Studies testing the hypothesized mechanism of action obtained from the connectivity map and use of the compound in heterospheroids and in vivo assays to further test its clinical potential will be the subject of future work. The novel and selective activity of furospinulosin 1 in this model of triple negative breast cancer makes it an interesting compound with strong therapeutic potential that merits further study.

## 4. Materials and Methods

### 4.1. Reagents

Samples for screening were obtained from the Harbor Branch Oceanographic Institute pure compound and enriched fraction libraries [43]. Stock solutions of samples were at a concentration of 1 mg/mL in methanol or ethanol. Methanol and ethanol used in the experiments were purchased from Fisher Scientific (Fair Lawn, NJ, USA). The 3-(4,5-Dimethyl-2-thiazolyl)-2,5-diphenyl-2H-tetrazolium bromide (MTT) used for cell viability assays was purchased from Sigma Chemical Co. (St. Louis, MO, USA). Matrigel was purchased from BD Biosciences (San Jose, CA, USA).

### 4.2. Cell Culture

The human triple negative breast cancer cell lines MDA-MB-231 (HTB-26) and MDA-MB-468 (HTB-132) were obtained from ATCC (Manassas, VA, USA), grown, aliquoted and maintained in liquid nitrogen. Aliquots were thawed and grown in DMEM medium (including 4 mM l-glutamine, 4.5 g/L glucose and 1.5 g/L sodium bicarbonate; ATCC 30-2002) supplemented with 10% fetal bovine serum (FBS; Hyclone SH3071, GE Healthcare/life sciences, Logan, UT, USA), 100 U/mL penicillin G sodium and 100 µg/mL streptomycin sulfate (Gibco, Carlsbad, CA, USA). Cells were maintained in a humidified incubator at 37 °C and 5% CO_2_. Cells were kept in culture for 10 weeks (20 passages) when a new aliquot was thawed.

### 4.3. 3D Spheroid Multiparametric Assay

A multiparametric live cell toxicity assay adapted from a previously published assay to measure cytotoxicity in spheroids [44] was chosen to measure cytotoxicity in 3D culture. This assay measures cell number based on nucleic acid staining with Hoechst 33342, induction of apoptosis based on a substrate that produces fluorescence upon cleavage of caspase 3/7 and viability using the cell impermeable dye 7-amino actinomycin D (7AAD) that will only label cells that have lost their cell membrane integrity (late apoptotic, necrotic, or dead cells). Compounds can induce regulated cell death in all its forms (apoptosis, regulated necrosis, caspase-free apoptosis, anoikis and autophagic cell death, to name a few) through many pathways (reviewed in [45]), and this assay provided us with the best chance of detecting the activity of the compound to induce cancer cell death, regardless of which pathway it activated to carry this out. The treatment time used in the previously published method was 48 h [44], and thus, this same time was used to test the HBOI compounds in this assay.

MDA-MB-231 or MDA-MB-468 cells were plated on a black plate with a clear-bottom low adherence spheroid 384-well tissue culture plate (Corning 3830; Corning, NY, USA) at a concentration of 1500 cells per well in ice-cold complete media without phenol red containing 2.5% matrigel (BD Biosciences, Billerica, MA, USA) in a final volume of 30 μL/well and incubated overnight. The next day, after confirming the formation of a spheroid, 30 μL of medium containing treatment at two times the final concentration was added. Enriched fraction library samples and pure compounds were tested at 5 μg/mL. Pure compounds that are known to be cytotoxic in the nanomolar range in 2D cultures were tested at 0.5 μg/mL. Other samples tested included 100 ng/mL super killer TRAIL and the known inducer of apoptosis microsclerodermin A at a concentration of 2.4 μg/mL (2× IC_50_ for inhibition of NFκB) [46]. Plate controls included 10 μM ABT737, 5 μg/mL 5-fluorouracil, 0.5 μg/mL doxorubicin, media alone and solvent controls. Cells were incubated with treatment for 48 h. At the end of this incubation, 20 μL/well of a staining mixture containing 2 drops/mL NucBlue Live Cell Stain Hoechst 33342 (Molecular Probes, Eugene, OR, USA), 5 μM CellEvent™ Caspase-3/7 Green Detection Reagent (Molecular Probes, Eugene, OR, USA) and 100 μg 7-aminoactinomycin D (7AAD; Sigma, St. Louis, MO, USA) were added and allowed to incubate for 3 h. Cells were fixed with 4% paraformaldehyde. Images were acquired using the ImageXpress^®^ Micro XLS Widefield High Content Analysis System (Molecular Devices, Sunnyvale, CA, USA), with a 10× Plan Fluor objective, binning at 2 and focusing on plate bottom, then offset by bottom thickness. A stack of 8 images separated by 10 μm starting at the well bottom and covering approximately the lower half of each spheroid were acquired. The best focus projection of this stack was analyzed using the Multi-Wavelength Cell Scoring Module of the MetaXpress 5.1.0.3 software (Molecular Devices, Sunnyvale, CA, USA). The results were plotted using Microsoft Excel (Redmond, WA, USA). To normalize results, the solvent control values were subtracted from the 7AAD and the Caspase 3 percent positive results for treatment samples. Total cell count was expressed as a percentage comparing treatments to respective solvent controls. Compounds were tested in duplicate within plates, and hits were confirmed by repeating the testing.

### 4.4. 2D Cell Viability Assay (MTT)

The MTT assay is the standard assay used to measure cytotoxicity in adherent cells in drug discovery [47]. This assay has been used extensively to report on the activity of many marine natural compounds discovered at HBOI. This assay is performed by seeding the cells and allowing them to adhere, then on the next day treating with compound for 72 h and assessing the viability of cells at the end of the experiment. This assay relies on the metabolization of the yellow 3-(4,5-dimethylthiazol-2-yl)-2,5-diphenyltetrazolium bromide (MTT) to its insoluble formazan, resulting in the formation of purple crystals. When cells lose their viability, they lose their ability to metabolize the compound, resulting in a marked change in color that is easy to measure using absorbance in a plate reader [47]. Thus, this standard assay and treatment time were used to test the compound for 2D cytotoxicity.

Cells were plated on a clear flat bottomed 384-well tissue culture plate at a concentration of 3000 cells per well in a volume of 30 μL/well and allowed to adhere overnight. At the end of this incubation, 30 μL of medium containing treatment at two times the final concentration was added. Enriched fraction library samples and pure compounds with low cytotoxicity (micromolar) were tested at 5 μg/mL. Pure compounds with known cytotoxicity at the nanomolar range were tested at 0.5 μg/mL. Other samples tested included 100 ng/mL super killer TRAIL and the known inducer of apoptosis microsclerodermin A at a concentration of 2.4 μg/mL. Plate controls included 10 μM ABT737, 5 μg/mL 5-fluorouracil, 0.5 μg/mL doxorubicin, media alone and solvent controls. The same compounds were tested in the 2D assay at the same concentrations used in the 3D assay. The cells were then incubated for 72 h at 37 °C and 5% CO_2_. After this incubation, 125 μg MTT was added to each well. The cells were then incubated for 3 h at 37 °C followed by centrifugation. The supernatant was removed and 100 μL acidified isopropyl alcohol (1:500 solution of hydrochloric acid to isopropanol) was added to each well to dissolve the crystals. The absorbencies of these solutions were measured at 570 nm with a plate reader (NOVOstar, BMG Labtech Inc., Durham, NC, USA). The resulting absorbencies were normalized against ethanol-treated cells using Microsoft Excel (Redmond, WA, USA).

### 4.5. IC_50_ Determination

The values for the percent decrease in cell number normalized against ethanol-treated cells were used to determine the dose needed to induce 50% cytotoxicity in the spheroid assay (IC_50_) using a non-linear regression curve fit with GraphPad Prism 5 software (GraphPad Software, San Diego, CA, USA).

### 4.6. Reverse Phase Protein Array (RPPA)

MDA-MB-231 cells were grown for 24 h as 2D adherent monolayers or as spheroids and then treated with ethanol (vehicle control) or 5 μg/mL furospinulosin 1 for 24 h (to avoid apoptosis). At the end of treatment, cells were harvested, and protein was isolated using RPPA lysis buffer (1% Triton × 100, 50 mM HEPES, pH 7.4, 150 mM NaCl, 1.5 mM MgCl_2_, 1 mM EGTA, 100 mM NaF, 10 mM Na pyrophosphate, 1 mM Na_3_VO_4_, 10% glycerol, protease and phosphatase inhibitors from Roche Applied Science Cat. # 05056489001 and 04906837001, respectively). Protein was adjusted to a concentration of 1.5 µg/µl and submitted to MD Anderson Reverse Phase Protein Array core. Samples were treated according to their published methods [48,49]. Briefly, samples were serial diluted and printed on nitrocellulose-coated slides. Slides were probed with 450 validated primary antibodies followed by detection with appropriate biotinylated secondary antibodies. The signal was detected by 3,3′-diaminobenzidine (DAB)-horse radish peroxidase (HRP) colorimetric reaction. Spot density was determined using Array-Pro Analyzer software (Media Cybernetics, Rockville, MD, USA), and protein concentration was determined by Super Curve Fitting. As part of the service, MDA Anderson provided a data report that included raw, normalized and median-centered data as well as a heat map. The protein from 3 independent experiments was examined in a single array, and the resulting linear normalized data were averaged, compared to a control and expressed as a percentage using Microsoft Excel.

### 4.7. Purification and Identification of Furospinulosin 1

Biological material: The marine sponge used in this study was identified as *Smenospongia cf. echina* [50] (Phylum Porifera, Class Demospongiae, Order Dictyoceratida, Family Thorectidae) [51] (Figure S5). The specimen was collected in the Eastern Gulf of Mexico near Pulley Ridge (latitude 26 15.785′ N; longitude 83 42.544′ W) using the Mohawk ROV operated by the University of North Carolina at a depth of 71 m (HBOI Sample ID 11-V-15-1-012). The sponge is yellow gray in color. The morphology is massive with 1–2 cm diameter oscula with smaller 2–3 mm clumped holes. The surface is smooth when alive and microconulose and grooved out of water. The skeleton has smooth amber color fibers that are primarily unpithed (40–60 µm).

Purification of furospinulosin 1: The sample was frozen immediately after collection and stored at −20 °C until extraction. An amount of 157 g of the frozen sponge was freeze-dried and then ground and extracted sequentially using a Dionex Accelerated Solvent Extractor in 3 steps (Step 1: heptane (yield equals 1.26 g); Step 2: CH_3_CH_2_OH–CH_3_CO_2_CH_2_CH_3_ 5:1 *v*/*v*, (yield equals 0.85 g); and Step 3: CH_3_OH:H_2_O 5:1 *v*/*v*, (yield equals 3.47 g)) at 100 °C with 3 static cycles per step. Amounts of 0.6 g of the heptane extract and 0.6 g of the CH_3_CH_2_OH–CH_3_CO_2_CH_2_CH_3_ (5:1 *v*/*v*) extract were combined and separated using an Isco Combiflash™ Rf4. The material was adsorbed onto Celite and solvent removed for solid sample loading. A 30 g RP Rf Gold C18 Column (Isco Teledyne, Lincoln, NE, USA) was used for the separation. The total run time was 32.3 column volumes (CV) over 23.8 min with a flow rate of 35 mL/minute. The column was eluted with gradient elution as follows: Solvent A: H_2_O, Solvent B: CH_3_CN, Solvent C: CH_3_OH; Solvent D: CH_2_Cl_2_; t = 0 min A:B (9:1 *v*/*v*) held for 3 CV then eluted with a linear gradient ending at 100% B over 12 CVs; held at 100% B for 5.3 CV; washed with 100% C for 3 CVs, then a linear gradient was conducted to 100% D over 3 CV and held at 100% D for 3 CV. The pressure was maintained at 200 psi over the course of the elution. Tubes 81–83 eluting between 23 and 24 CV were combined and after solvent removal yielded 22.6 mg of a fraction containing furospinulosin 1 as a major component. An amount of 8.9 mg of the fraction was further purified by preparative HPLC using a Vydac C-18 protein and peptide column (19 × 150 mm, 10 µ particle size) using gradient elution (Solvent A: H_2_O:CH_3_CN (95:5 *v*/*v*); Solvent B: CH_3_CN; flow rate 12 mL/min t = 0 min. A:B 50:50, t = 6 min 100% B, t = 20 min 100% B). Furospinulosin 1 eluted at 13.4 min with a yield of 2.9 mg was used in the biological studies. Interpretation of the full 1D and 2D NMR data set for the active compound and comparison to published values identified the active compound as furospinulosin 1 [37] (Table S1, Figures S1–S4). 

## Data Availability

Any data that support this manuscript that are not included in the manuscript or supplementary materials are available upon reasonable request to the corresponding author.

## Supplementary Materials

The following are available online at https://www.mdpi.com/article/10.3390/md19050249/s1, Figure S1. Structure of furospinulosin 1 with numbering, chemical shifts and COSY or HMBC correlations. Table S1. NMR data for furospinulosin 1 (d_4_-methanol 600 MHz). Figure S2. ^1^H NMR spectrum of furospinulosin 1 in *d*_4_-methanol (600 MHz) with assignments. Figure S3. ^13^C NMR of furospinulosin 1 in *d_4_*-methanol (150 MHz) with assignments. Figure S4. DART Mass Spectrum of furospinulosin 1. Figure S5. Taxonomy of the specimen used in this study. Table S2. Function for proteins most changed when comparing vehicle control treated 2D against vehicle control treated 3D cells. Table S3. Function for proteins most changed when comparing vehicle control treated 2D cells against furospinulosin 1 treated 2D cells. Table S4. Function for proteins most changed when comparing vehicle control treated 3D cells against furospinulosin 1 treated 3D cells. Figure S6. Effects of furospinulosin 1 on MEK signaling pathway. Figure S7. STRING Network of Biologically Connected Proteins from the subset of proteins that changed more than 25% in 3D-treated cells.
