# Supplementary material for: The Marine Natural Product Furospinulosin 1 Induces Apoptosis in MDA-MB-231 Triple Negative Breast Cancer Cell Spheroids, But Not in Cells Grown Traditionally with Longer Treatment
Source: Mar Drugs. 2021 Apr 28;19(5):249. doi: 10.3390/md19050249 (PMC8145321; doi:10.3390/md19050249)

The Marine Natural Product Furospinulosin 1 Induces Apoptosis in MDA-MB-231 Triple Negative Breast Cancer Cell Spheroids, but Not When Cells Are Grown Traditionally.

Esther A. Guzmán•, Tara P. Pitts, Priscilla L. Winder, Amy E. Wright.

Marine Biomedical and Biotechnology Research, Harbor Branch Oceanographic Institute at Florida Atlantic University, 5600 US 1 North, Ft. Pierce, Florida, 34946, USA.

Figure S1. Structure of furospinulosin 1 with numbering, chemical shifts and COSY or HMBC correlations.

Table S1. NMR data for furospinulosin 1 (d_4-methanol 600 MHz)

Figure S2. ^{1}H NMR spectrum of furospinulosin 1 in d_4-methanol (600 MHz) with assignments.

Figure S3. ^{13}C NMR of Furospinulosin 1 (d_4-methanol 150 MHz) with assignments.

Figure S4. DART Mass Spectrum of furospinulosin 1

Figure S5. Taxonomy of the specimen used in this study

Table S2. Function for proteins most changed when comparing vehicle control treated 2D against vehicle control treated 3D cells.

Table S3. Function for proteins most changed when comparing vehicle control treated 2D cells against furospinulosin 1 treated 2D cells.

Table S4. Function for proteins most changed when comparing vehicle control treated 3D cells against furospinulosin 1 treated 3D cells.

Figure S6. Effects of Furospinulosin 1 on MEK Signaling Pathway.

Figure S7. STRING Network of Biologically Connected Proteins from the subset of proteins that changed more than 25% in 3D treated cells.

Figure S1. Structure of furospinulosin 1 with numbering, chemical shifts and COSY or HMBC correlations.

Selected Correlations observed in the 2D-^{1}H-^{1}H-*g*COSY spectrum

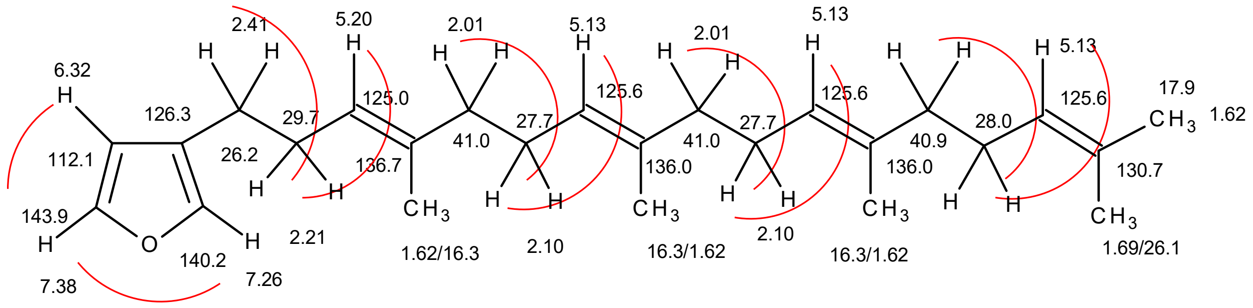

Selected Correlations observed in the 2D-edited g-HMBC spectrum optimized for J=8 Hz

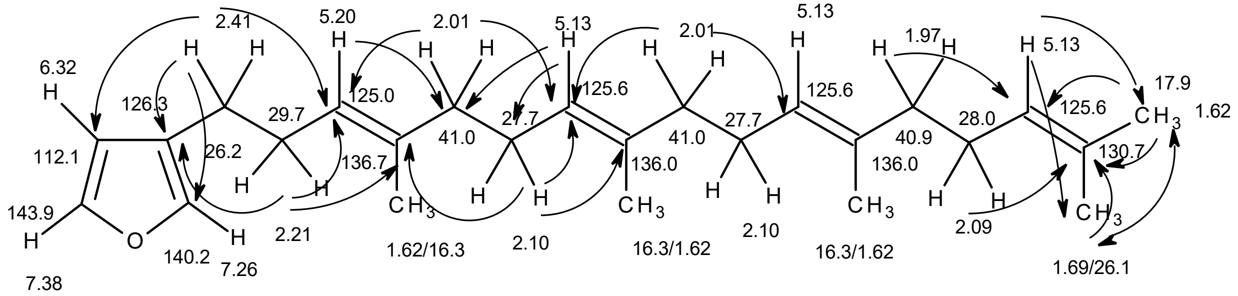

Numbering of Furospinulosin 1

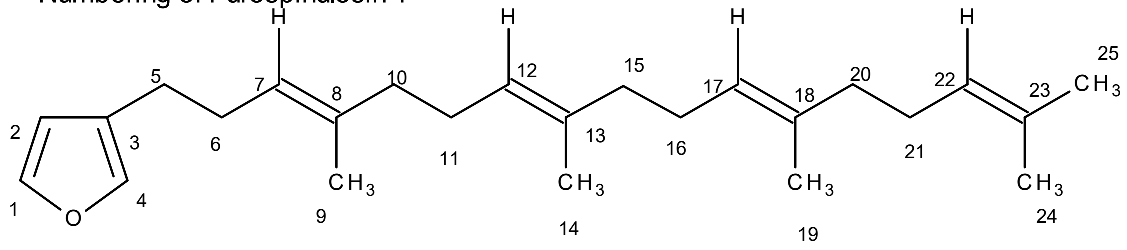

Table S1. NMR data for furospinulosin 1 (d_4-methanol 600 MHz)

Atom No.	^{13}C		^{1}H	Mult (*J* Hz)	COSY	HMBC[ab]
**1**	143.9	CH	7.38	s	2, 4	2,3,4
**2**	112.1	CH	6.32	s	1,4	1,3.4
**3**	126.3	q-C				
**4**	140.2	CH	7.26	s	1, 2, 5	1,2,3
**5**	26.2	CH_2	2.41	2H, t (7.5)	4, 6	2,3,4.6
**6**	29.7	CH_2	2.21	2H, q (7.6)	5, 7, 10	5,7,8
**7**	125.0	CH	5.2	t (6.9)	6, 9, 10	6,9,10
**8**	136.7	q-C				
**9**	16.3	CH_3	1.62	3H, s	7	8,10
**10**	41.0*	CH_2	2.01	2H, m	7, 11	8,9,11
**11**	27.7	CH_2	2.1	2H, m	10, 12	10,12,13
**12**	125.6	CH	5.13	m	11, 14	
**13**	136.0	q-C				
**14**	16.3	CH_3	1.62	3H, s	12	12,13,15
**15**	41.0*	CH_2	2.01	2H, m	16	12 and or 17, 14, 16
**16**	27.7	CH_2	2.1	2H, m	15,17	13 and or 18, 15
**17**	125.6	CH	5.13	m	16, 19	15 and or 20, 19
**18**	136.0	q-C				
**19**	16.3	CH_3	1.62	3H, s	17	17, 18, 20
**20**	40.9*	CH_2	2.01	2H, m	21	18, 19, 21
**21**	28.0	CH_2	2.09	2H, m	20, 22, 24, 25	20, 23
**22**	125.6	CH	5.13	m	21	30,24
**23**	130.7	q-C				
**24**	26.1	CH_3	1.69	3H, s	21	22, 23, 25
**25**	17.9	CH_3	1.62	3H, s	21	22, 23, 24
	[a] there is substantial overlap in the chemical shifts for the isoprene units and the HMBC correlations reported are assigned based upon the numbering for adjacent isoprene functionality.					
	[b] correlations are from the proton to the carbons listed					
	* assignments interchangeable					

Figure S2. ^{1}H NMR spectrum of furospinulosin 1 in d_4-methanol (600 MHz) with assignments

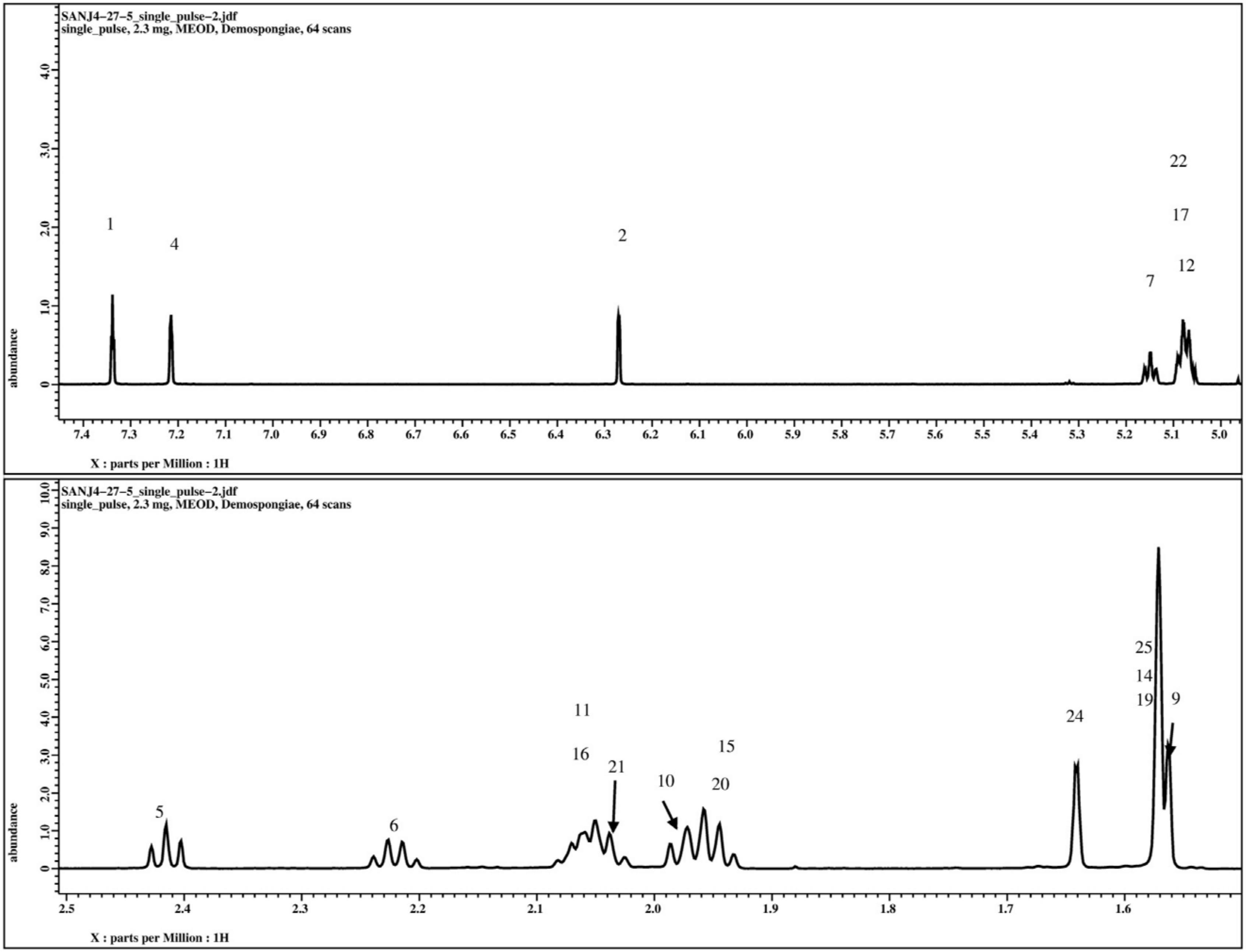

Figure S2. ^{13}C NMR of Furospinulosin 1 (d_4-methanol 150 MHz) with assignments.

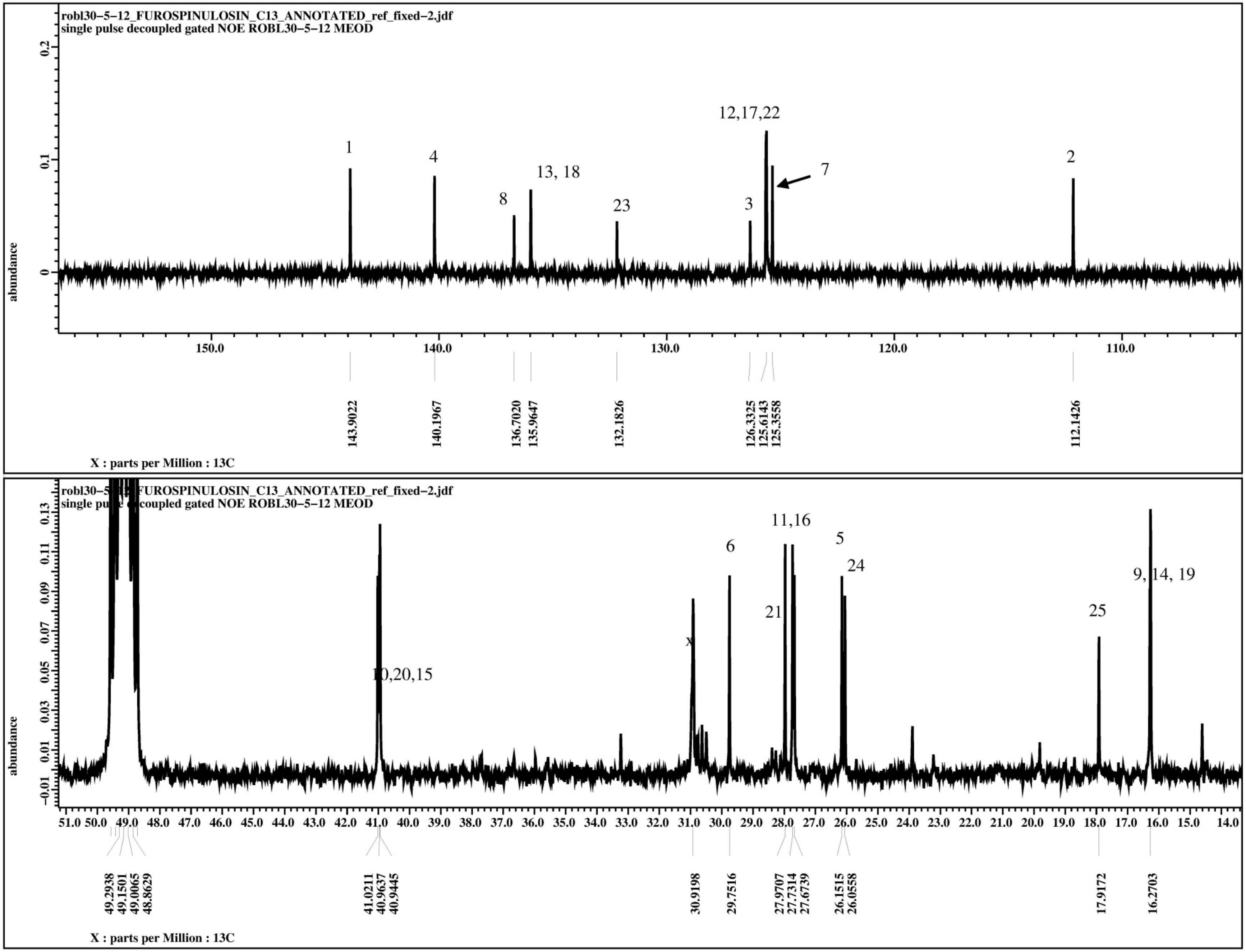

Figure S4. Mass Spectrum of furospinulosin 1.

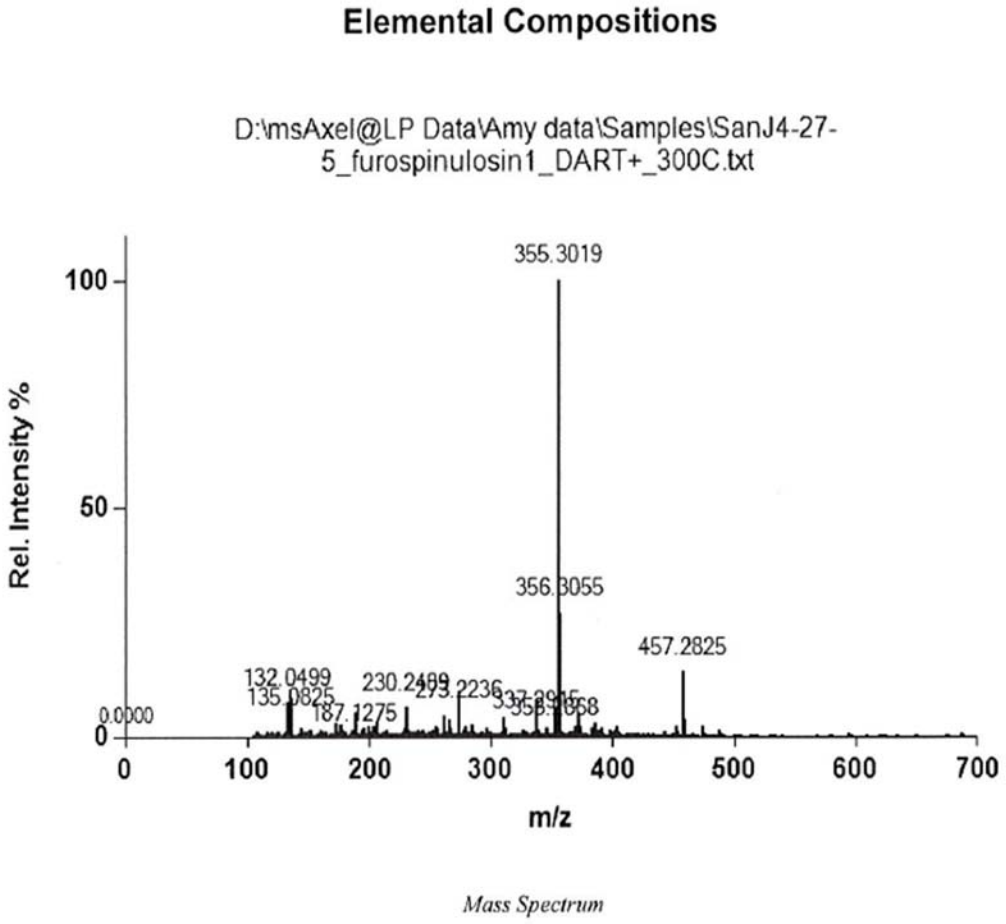

HRMS detected by DART ionization in positive ion mode on a JEOL AccuTOF. DART probe temperature 300C; ion guide 1000

$[M+H]^+$ *m/z* observed 355.3019, calc for C_{25} H_{39} O_1 355.3001

Elemental Compositions
Element Limits: C 0/50 H 0/100 O 0/10 N 0/10
Tolerance: 5 mmuEven or odd electron ion or both: Even
Electron correction: None.Charges: 1
Minimum unsaturation: -1Maximum unsaturation: 100

Calc. m/z	Abund %	mmu	DBE	Composition
355.304613	100.000	2.73	3.5	C15H35N1O
355.300091	100.000	-1.79	6.5	C25H39O1

S5. Taxonomy of the specimen used in this study

The marine sponge used in this study was identified as *Smenospongia* cf. *echina* (Laubenfels, 1934) [Phylum Porifera, Class Demospongiae, Order Dictyoceratida, Family Thorectidae] The specimen was collected in the eastern Gulf of Mexico near Pulley Ridge (latitude 26 15.785' N longitude 83 42.544'W] using the Mohawk ROV operated by the University of North Carolina at a depth of 71 m (HBOI Sample ID 11-V-15-1-012).

The sponge is yellow gray in color. The morphology is massive with 1-2 cm diameter oscula with smaller 2-3 mm clumped holes. The surface is smooth when alive and microconulose and grooved out of water. The skeleton has smooth amber color fibers that are primarily unpithed (40- 60 μm).

Deck and In Situ photos of Sample 11-V-15-1-012 used in the purification of furospinulosin 1.

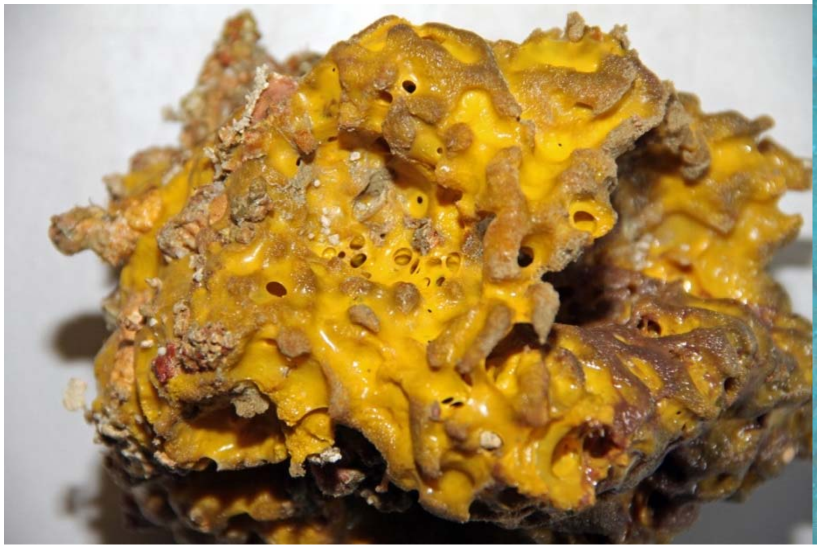

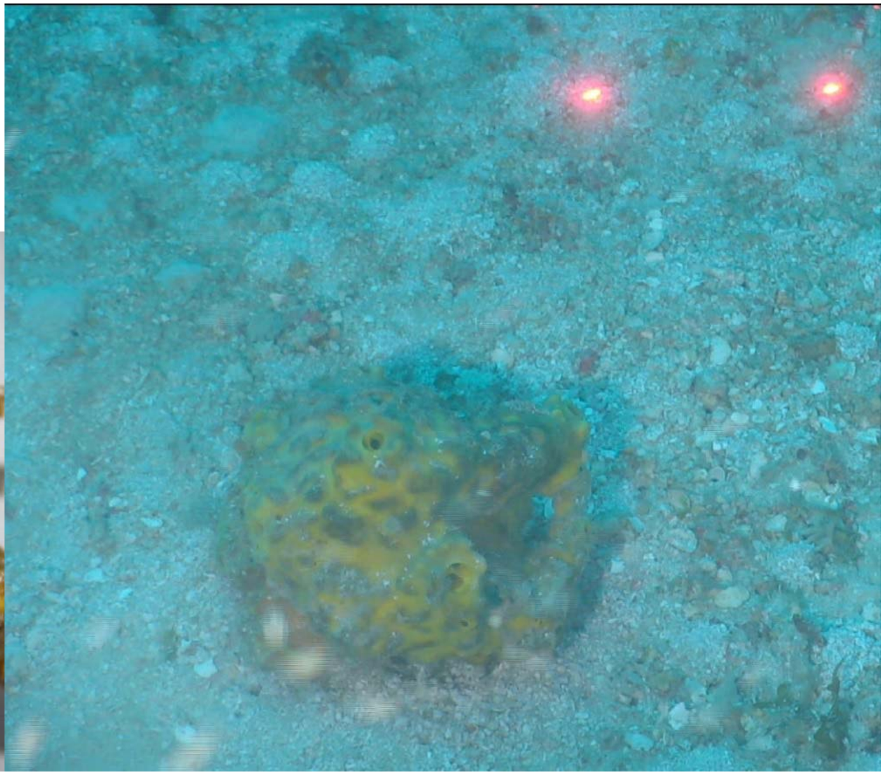

Rützler, K.; Piantoni, C.; Van Soest, R.W.M.; Díaz, M.C. (2014). Diversity of sponges (Porifera) from cryptic habitats on the Belize barrier reef near Carrie Bow Cay. *Zootaxa.* 3805(1): 1-129., *available online at* https://doi.org/10.11646/zootaxa.3805.1.1

http://www.marinespecies.org/aphia.php?p=taxdetails&id=574343

Table S2. Function for proteins most changed when comparing vehicle control treated 2D against vehicle control treated 3D cells.

Protein Changes Based on How Cells Were Grown (2D VC vs 3D VC)		
**Antibody Name**	**Percent Change**	**Protein Function**
Notch1-cleaved	-62 ± 2	Notch proteins regulate cell-fate determination. Activity is regulated through sequential cleavage steps.
PAR	-61 ± 25	poly(ADP-ribose) (PAR) polymer is a product of poly(ADP-ribose) polymerase (PARP) activity that may serve as a signal for cell death. PARP is a nuclear enzyme that is important in DNA repair.
EVI1	-52 ± 3	Evi-1 (Ecotropic virus integration site 1) is a zinc finger transcription factor implicated in solid tumor formation.
EphA2	-46 ± 5	EphA2 is a receptor tyrosine kinase (RTK) that binds the glycosylphosphatidylinositol-anchored ephrin A-1 ligand. EPHA2 is overexpressed in many cancer types where its thought to participate in crosstalk between the RAS-PI3K -AKT and RAS-MAPK signaling pathways.
YAP_pS127	-44 ± 6	Yes-associated protein (YAP) is a 14-3-3-binding molecule that binds to the SH3 domain of the tyrosine kinase Yes. 14-3-3 proteins regulate differentiation, cell cycle progression and apoptosis. YAP is thought to link events at the plasma membrane and cytoskeleton to inhibition of transcription in the nucleus.
p70-S6K_pT389	169 ± 5	p70 S6 kinase is a mitogen activated Ser/Thr protein kinase needed for cell growth and G1 cell cycle progression. Phosphorylation of Thr389 is necessary for activation.
DNA-Ligase-IV	156 ± 30	DNA ligase that joins single-strand breaks in an ATP-dependent reaction. One of the essential proteins for DNA repair of DNA double-strand breaks through the non-homologous end joining (NHEJ) pathway .
PEA-15_pS116	89 ± 26	Proliferation and apoptosis adaptor protein 15 (PEA-15) regulates apoptosis, cell growth and glucose use. Phosphorylation at Ser 116 and Ser 104 regulates its ability to bind the Fas Associated Death Domain (FADD) and the Extracellular Receptor Kinase (ERK).
PDGFR-b	82 ± 16	Platelet derived growth factor receptor β is a receptor tyrosine kinase that regulates cell growth, actin reorganization, migration and differentiation.
Akt1_pS473	76 ± 12	through phosphorylation and inactivation of Bad and caspase 9 among others. It regulates glycogen synthesis through phosphorylation and inactivation of the glycogen synthase kinase 3 α and β. Phosphorylation of AKT1 at ser 473 by the mammalian target of rapamycin (mTOR) facilitates Akt 1 phosphorylation at Thr 308. Both events are needed for AKT1 activation.

Table S3. Function for proteins most changed when comparing vehicle control treated 2D cells against furospinulosin 1 treated 2D cells

**Protein Changes Based on Treatment 2D (2D VC vs 2D treated)**		
**Antibody Name**	**Percent Change**	**Protein Function**
VHL-EPPK1	-68 ± 2	The von Hippel-Lindau (VHL) protein binds to the transcription factore Elongin subunits B and C, preventing its association with subunit A and thus transcription. VHL is a negative regulator of cellular growth.
HER2	-44 ± 5	The epidermal growth factor receptor 2 (Her2,c-erbB-2) is a receptor tyrosine kinase that requires a co-receptor for ligand binding. It regulates the growth and stabilization of microtubules through its inhibition of GSK3β.
GATA3	-43 ± 3	GATA proteins are a family of transcription factors that bind to the DNA sequence *GATA* . GATA3 is essential in the development of luminal breast epithelium, as well as the development of other tissues and T cells.
DM-K9-Histone-H3	-37 ± 13	Histone H3 activates or inhibits transcription. It may also help maintain a specific chromatin structure. Methylation at lysine 9 correlates with transcriptional repression and serves as a specific binding site for heterochromatin protein 1 (HP1).
14-3-3-epsilon	-35 ± 10	14-3-3s are multi-functional proteins that regulate signaling pathways such as apoptosis, cell cycle progression, autophagy, glucose metabolism, and cell motility by binding target proteins at specific phosphorylation sites.
RRM1	34 ± 5	[illegible] enzyme that catalyzes the rate-limiting step in the synthesis of deoxynucleotide triphosphates (dNTPs).
Mitofusin-1	31 ± 18	Mitofusin-1 and -2 are mitochondrial transmembrane GTPases that work together to to regulate mitochondrial fusion.
IGFBP3	30 ± 15	Insulin-like growth factor-binding protein 3 binds the insulin-like growth factors IGF-1 and IGF-2 preventing them from activating the IGF1R and thus is considered to have antiproliferative effects. However, in certain cells it can promote growth by transactivating the EGFR.
XIAP	24 ± 8	The X-linked inhibitor of apoptosis protein (XIAP) stops programmed cell death (apoptosis) from occurring by binding caspases 3,7, and 9 blocking them from binding their substrates.
Chk1	22 ± 11	The Checkpoint kinase 1 activates the DNA damage response stopping the cell cycle and allowing a cell to undergo DNA damage repair or start apoptotic signaling to prevent cells with damaged DNA from proliferating.

Table S4. Function for proteins most changed when comparing vehicle control treated 3D cells against furospinulosin 1 treated 3D cells

Protein Changes Based on Treatment 3D (3D VC vs 3D treated)		
**Antibody Name**	**Percent Change**	**Protein Function**
Akt	-41 ± 6	The Ser/Thr protein kinase Akt is also known as the protein kinase B (PKB). AKT1 promotes survival through phosphorylation and inactivation of Bad and caspase 9 among others. It regulates glycogen synthesis through phosphorylation and inactivation of the glycogen synthase kinase 3 α and β.
DUSP4	-38 ± 8	Dual specificity protein phosphatase 4 inactivates ERK1, ERK2 and JNK through dephosphorylation, thus negatively regulating cell proliferation
VHL-EPPK1	-37 ± 5	The von Hippel-Lindau (VHL) protein binds to the transcription factore Elongin subunits B and C, preventing its association with subunit A and thus transcription. VHL is a negative regulator of cellular growth.
PDH	-36 ± 3	Pyruvate dehydrogenase catalyzes an important step in the citric acid cycle necessary for cellular respiration.
MMP14	-34 ± 7	Membrane-type member of the matrix metalloproteinase family (MMP) that break down extracellular matrix during normal physiological functions. MMP14 is thought to activate MMP2, and through this activation contribute to carcinogenesis.
Stathmin-1	65 ± 2	Regulates the cell cycle and microtubule dynamics. Considered an oncoprotein as it can lead to uncontrolled cell proliferation if dysfunctional.
EMA	64 ± 6	The epithelial membrane antigen is a mucin known to bind pathogens and to stimulate signal transduction by binding p53.
Mitofusin-2	46 ± 18	Mitofusin-1 and -2 are mitochondrial transmembrane GTPases that work together to to regulate mitochondrial fusion.
NDUFB4	44 ± 20	transfers electrons from the reduced form of the nicotinamide adenine dinucleotide (NADH) to ubiquinone.
PRAS40_pT246	40 ± 5	This 40 kDa proline-rich protein (PRAS40) is a substrate of Akt that binds 14-3-3 proteins and mTOR. Binding leads to the inhibition of mTOR signaling. Phosphorylation of PRAS40 at Thr246 facilitates mTOR signaling activation, although other events are necessary to fully activate mTOR.

Figure S6. Effects of Furospinulosin 1 on MEK Signaling Pathway.

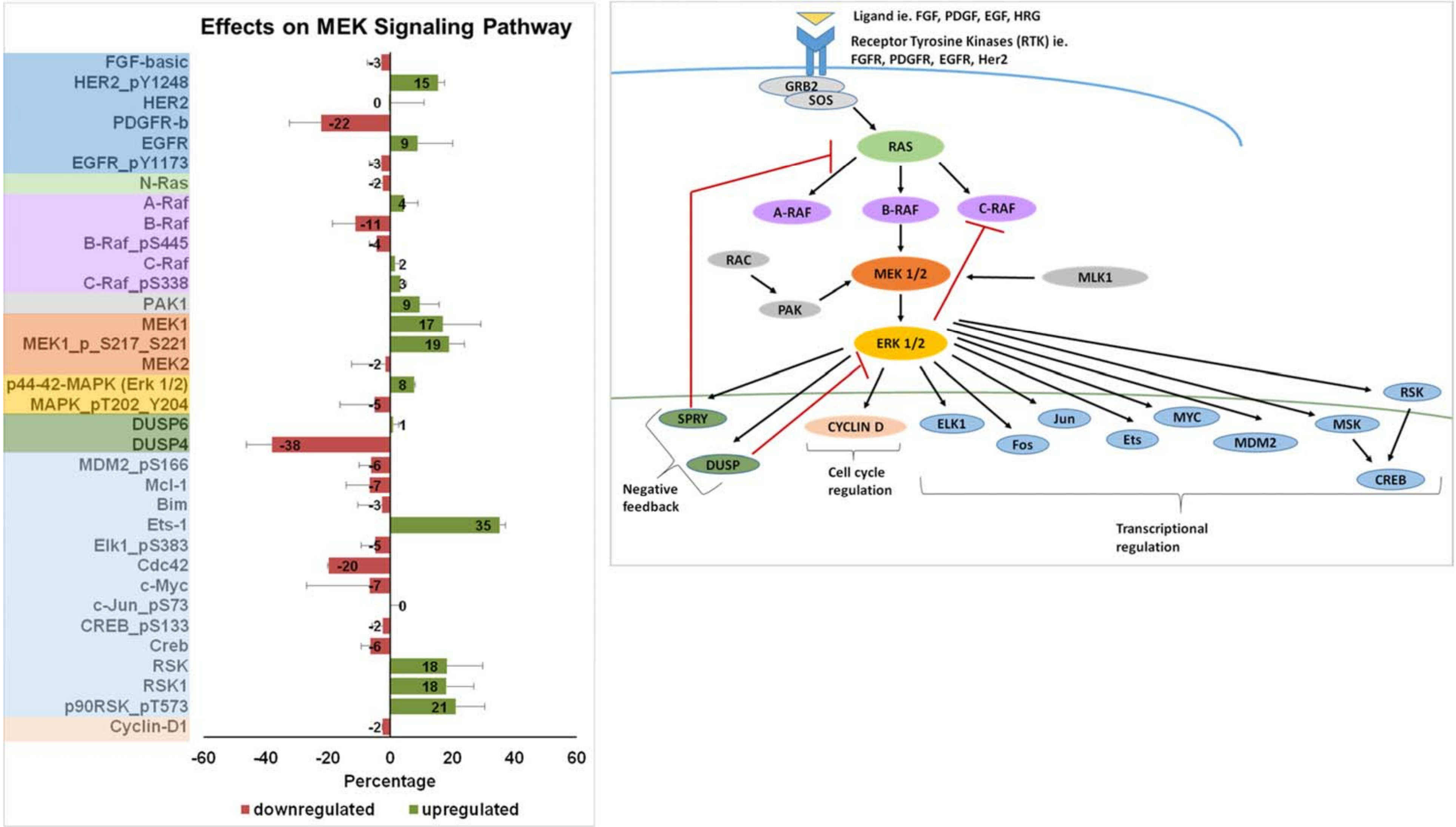

Figure S6. Effects of Furospinulosin 1 on MEK Signaling Pathway. Analysis of RPPA results through the Compare Algorithm from the Broad Institute had suggested MEK inhibition as a potential mode of action for furospinulosin 1. This prompted a closer look at the proteins included in the array that correspond to this pathway. While there are proteins involved in transcriptional regulation downstream of phosphorylated Erk 1/2 that are downregulated, not all of the proteins downstream are. Furthermore, one of the most downregulated is DUSP4 which is part of the negative feedback of Erk 1/2, and downregulation should lead to Erk 1/2 being more active. Therefore, further investigation is needed to confirm that inhibition of MEK is the mode of action of furospinulosin 1.

Figure S7. STRING Network of Biologically Connected Proteins from the subset of proteins that changed more than 25% in 3D treated cells.

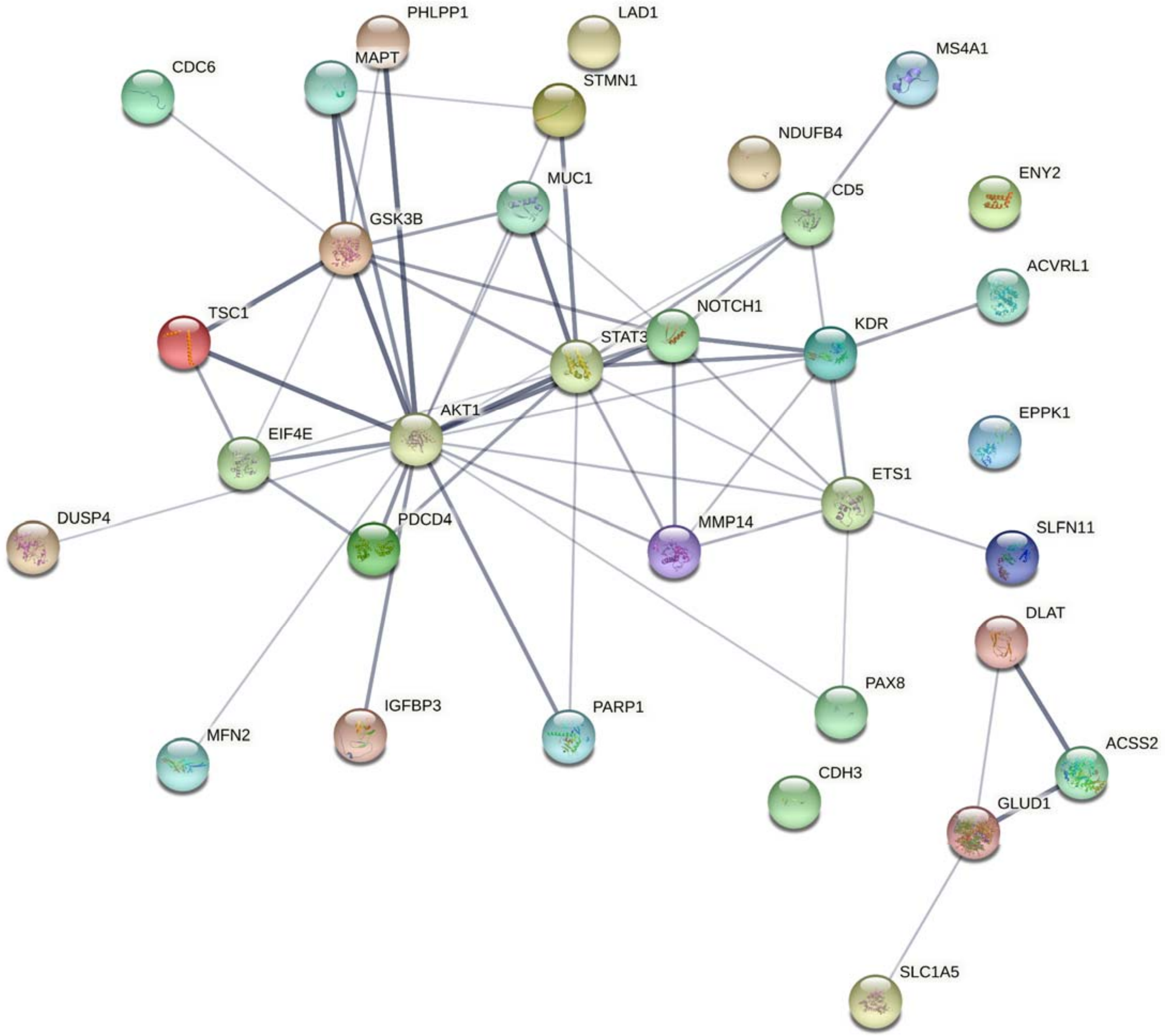

Supplement: Supplementary file 1 [file marinedrugs-19-00249-s001.zip › marinedrugs-1160843-supplementary.pdf]
